# P-1182. In Vitro Activity of Omadacycline and Comparator Agents Against Periodontal Pathogens

**DOI:** 10.1093/ofid/ofaf695.1375

**Published:** 2026-01-11

**Authors:** Courtenay J Kelley, Angela K Mendez, Mary Thwaites, Christopher M Pillar, Diane M Anastasiou, Alisa W Serio, David A Hufnagel

**Affiliations:** Microbiologics, Portage, Michigan; Microbiologics, Portage, Michigan; microbiologics, kalamazoo, Michigan; Microbiologics, Portage, Michigan; Paratek Pharmaceuticals, Inc., King of Prussia, PA; Paratek Pharmaceuticals, Inc., King of Prussia, PA; Microbiologics, Portage, Michigan

## Abstract

**Background:**

Omadacycline is a tetracycline class aminomethylcycline antibiotic FDA-approved for the treatment of community-acquired bacterial pneumonia and acute bacterial skin and skin structure infections in adults (IV or oral). Omadacycline has broad spectrum antibacterial activity against Gram-positive and -negative bacteria, including aerobic, anaerobic and atypical bacteria, and has demonstrated immunomodulatory properties. The objective of this study was to evaluate omadacycline activity against common periodontal pathogens. Severe periodontal disease, inflammation of tissues around the teeth, is estimated by the World Health Organization to affect more than a billion people worldwide. In certain cases, antibiotics are prescribed as adjunct therapy to minimize infection and decrease inflammation, with the most common being tetracyclines, metronidazole, fluoroquinolones, amoxicillin, and macrolides.

**Figure:**
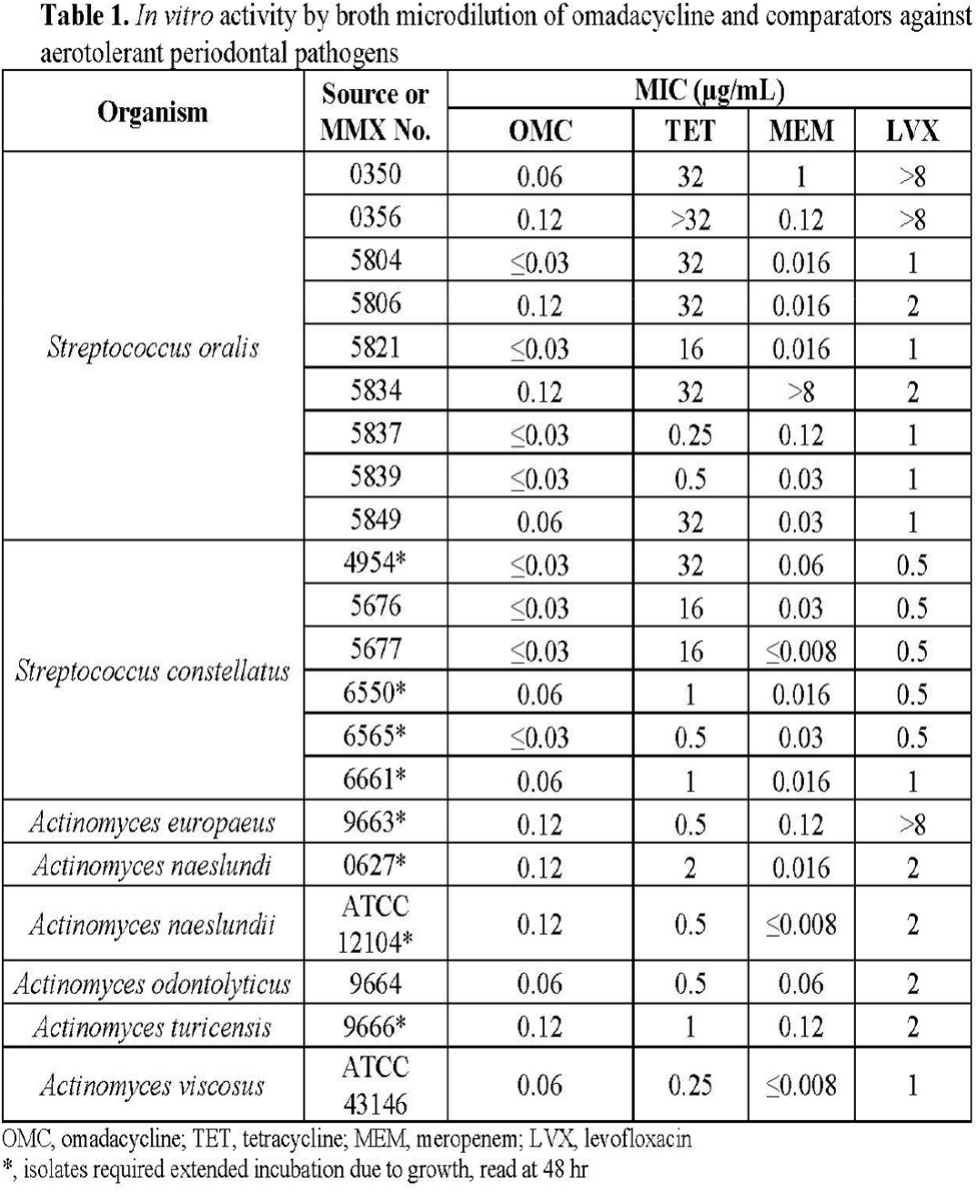

**Figure:**
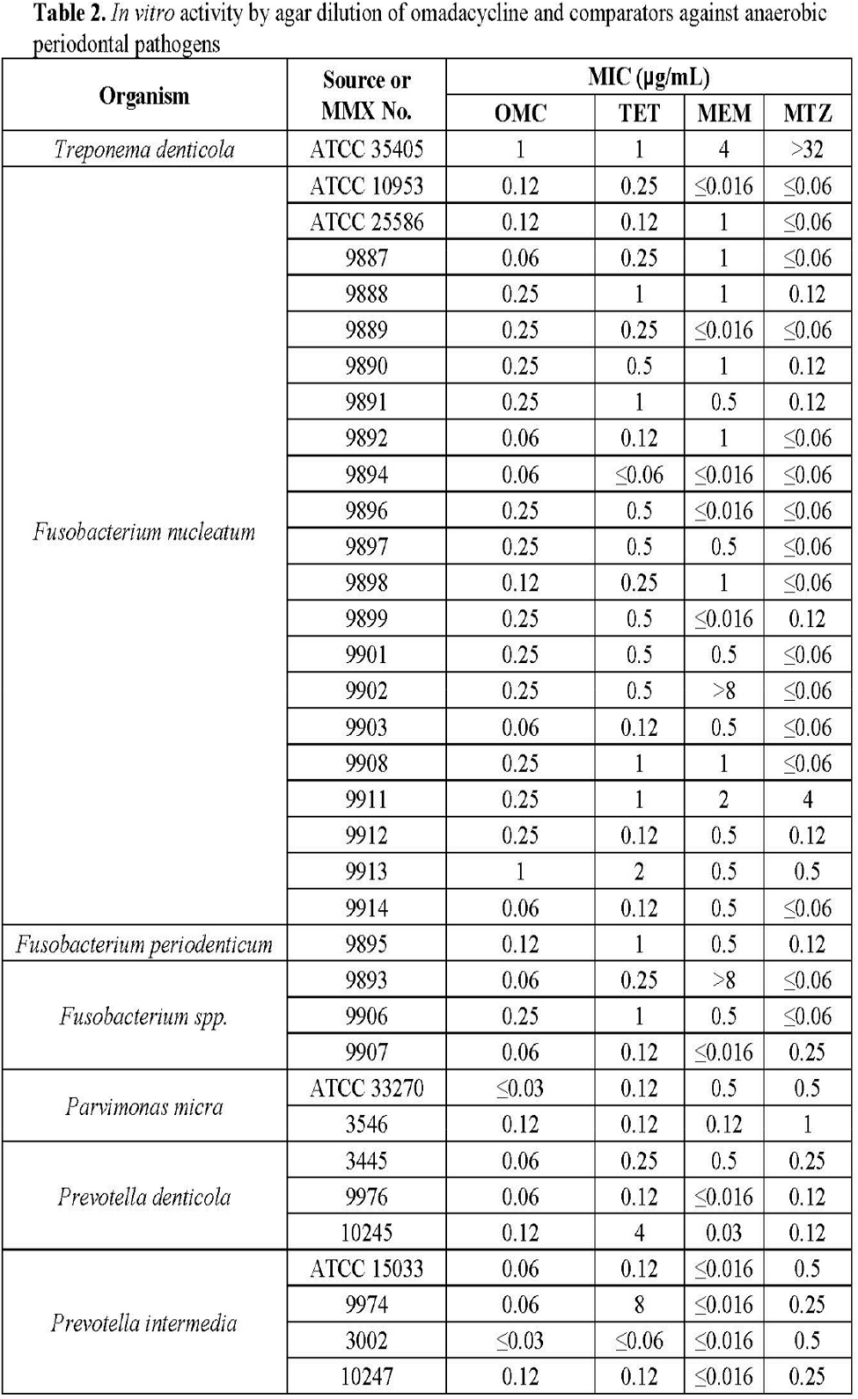

**Methods:**

Broth microdilution was performed according to CLSI against aerotolerant streptococci and *Actinomyces* spp. that were able to grow in 5% CO_2_ atmosphere. Agar dilution was performed according to CLSI for anaerobes. Omadacycline and comparators tetracycline, meropenem, levofloxacin and metronidazole were evaluated against 63 clinical isolates collected from New York, Indiana, Illinois, and California from 2009 to 2018 and 10 ATCC strains. In total, 73 isolates were evaluated across 8 genera: *Streptococcus, Actinomyces, Treponema, Fusobacterium, Parvimonas, Prevotella, Peptostreptococcus,* and *Aggregatibacter*.

**Figure:**
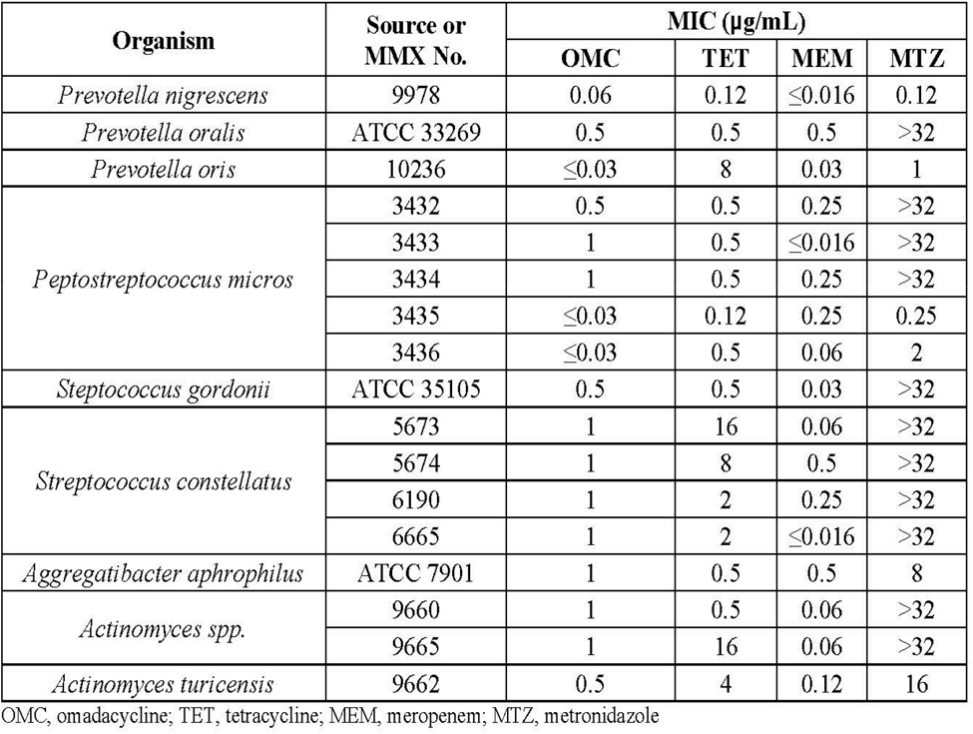

**Figure:**
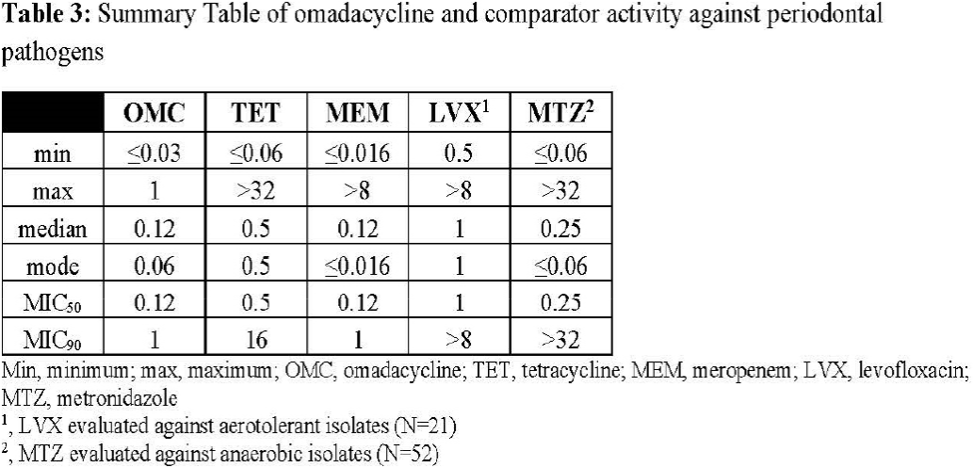

**Results:**

Omadacycline MIC values ranged from ≤0.03 to 1 µg/mL with median MIC, MIC_50_, and MIC_90_ values of 0.12, 0.12, and 1 µg/mL, respectively, against all isolates tested. The omadacycline median MIC, MIC_50_, and MIC_90_ results were identical to those of meropenem and lower than those of tetracycline and levofloxacin. Tables 1 - 3.

**Conclusion:**

Omadacycline demonstrated potent *in vitro* activity against a diverse collection of periodontal disease pathogens. These *in vitro* data, combined with other attributes of omadacycline, including immunomodulatory properties, warrant further investigation of omadacycline targeting periodontitis.

**Disclosures:**

Diane M. Anastasiou, BS, Paratek Pharmaceuticals, Inc.: Employee Alisa W. Serio, PhD, Paratek Pharmaceuticals, Inc.: Employee

